# Targeted removal of the FA2 site on human albumin prevents fatty acid–mediated inhibition of Zn^2+^ binding

**DOI:** 10.1016/j.jlr.2024.100560

**Published:** 2024-05-14

**Authors:** Dongmei Wu, Stephen J. Hierons, Sirilata Polepalli, Michal Gucwa, Remi Fritzen, Michal Markiewicz, Juan Sabín, Wladek Minor, Krzysztof Murzyn, Claudia A. Blindauer, Alan J. Stewart

**Affiliations:** 1 School of Medicine, University of St. Andrews, St. Andrews, UK; 2 Department of Chemistry, University of Warwick, Coventry, UK; 3 Department of Computational Biophysics and Bioinformatics, Jagiellonian University, Krakow, Poland; 4 AFFINImeter Scientific Team, Software 4 Science Developments, Santiago de Compostela, Spain; 5 Applied Physics Department, University of Santiago de Compostela, Santiago de Compostela, Spain; 6 Department of Molecular Physiology and Biological Physics, University of Virginia School of Medicine, Charlottesville, VA, USA

**Keywords:** ^13^C-NMR, allosteric interaction, free fatty acids, isothermal titration calorimetry, serum albumin, zinc

## Abstract

Zinc is required for virtually all biological processes. In plasma, Zn^2+^ is predominantly transported by human serum albumin (HSA), which possesses two Zn^2+^-binding sites of differing affinities (sites A and B). Fatty acids (FAs) are also transported by HSA, with seven structurally characterized FA-binding sites (named FA1-FA7) known. FA binding inhibits Zn^2+^-HSA interactions, in a manner that can impact upon hemostasis and cellular zinc uptake, but the degree to which binding at specific FA sites contributes to this inhibition is unclear. Wild-type HSA and H9A, H67A, H247A, and Y150F/R257A/S287A (FA2-KO) mutant albumins were expressed in *Pichia pastoris*. Isothermal titration calorimetry studies revealed that the Zn^2+^-binding capacity at the high-affinity Zn^2+^ site (site A) was reduced in H67A and H247A mutants, with site B less affected. The H9A mutation decreased Zn^2+^ binding at the lower-affinity site, establishing His9 as a site B ligand. Zn^2+^ binding to HSA and H9A was compromised by palmitate, consistent with FA binding affecting site A. ^13^C-NMR experiments confirmed that the FA2-KO mutations prohibited FA binding at site FA2. Zn^2+^ binding to the FA2-KO mutant was unaffected by myristate, suggesting binding at FA2 is solely responsible for inhibition. Molecular dynamics studies identified the steric obstruction exerted by bound FA in site FA2, which impedes the conformational change from open (FA-loaded) to closed (FA-free) states, required for Zn^2+^ to bind at site A. The successful targeting of the FA2 site will aid functional studies exploring the interplay between circulating FA levels and plasma Zn^2+^ speciation in health and disease.

## Graphical abstract

**Figure:**
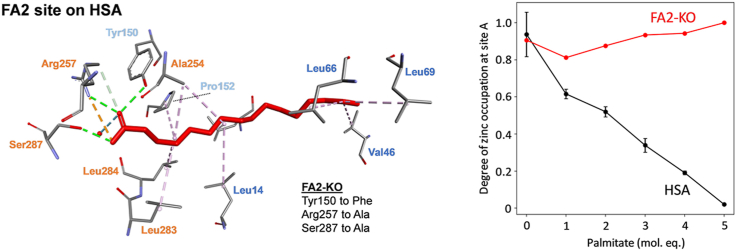

Zinc is an essential element required for many important biological processes. In plasma, Zn^2+^ is mainly bound and transported by human serum albumin (HSA) (1), a single chain protein composed of three homologous domains (I–III), each split into two subdomains (A and B). This interaction serves two purposes: Firstly, it enables Zn^2+^ to be distributed throughout the body (2), and secondly, it allows HSA to act as a buffer to help control Zn^2+^-mediated processes within the circulation. These include the regulation of coagulation (3, 4), insulin dynamics (5, 6), and cellular Zn^2+^ uptake (7). Two Zn^2+^-binding sites on equine serum albumin (ESA) have been identified and structurally characterized, both located between domains I and II (Fig. 1A) (8). The primary site (also known as site A) is formed by His67, His247, and Asp249 sidechains. A secondary site (known as site B), which binds Zn^2+^ with lower affinity, involves coordination to sidechain groups from His9, Asp13, and Asp255.

**Fig. 1:**
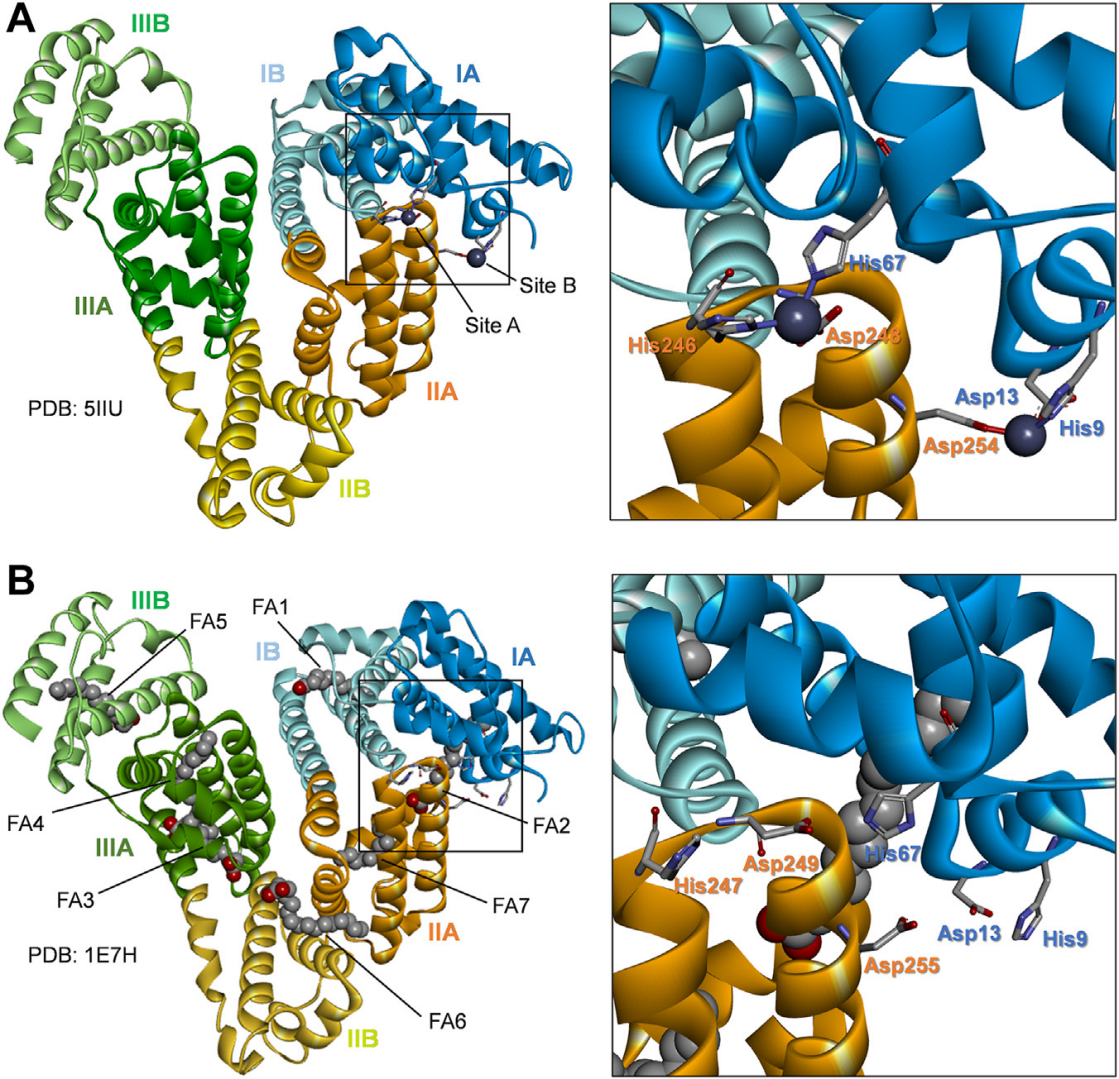
Structure of serum albumins showing locations of Zn^2+^ and FA-binding sites. A: Structure of equine albumin complexed with Zn^2+^ (PDB: 5IIU) (8) showing locations of conserved Zn^2+^ sites A and B. B: Structure of human albumin complexed with palmitate (1E7H) (9). The seven FA-binding sites (FA1–7) are shown. Zoomed images show locations of Zn^2+^-coordinating sidechains.

Albumin is also the primary transporter of fatty acids (FAs). FAs have been shown to compromise the Zn^2+^-binding capacity of albumin (4, 10). Insights from the crystal structures of apo-, Zn^2+^-bound, and FA-bound albumins have suggested that the effect of FAs on Zn^2+^ binding is mediated by an allosteric mechanism involving the domain I/II interface (2, 4, 10). Albumin has seven FA-binding sites (FA1–FA7; Fig. 1B) (9). Of these sites, the high-affinity FA2 site, is in closest proximity to site A. Like site A and B, FA2 sits at the interface between domains I and II and does not appear to be fully preformed in FA-free HSA (4, 11). Accommodation of a FA molecule at FA2 requires the convergence of two half-sites dislocated from each other by approximately 10 Å. A substantial rotation of domain I relative to domain II is required to bring them together. These changes also alter the architecture of site A significantly. In FA-bound albumin structures, His67 (domain I) is shifted by approximately 8 Å away from His247 and Asp249 (domain II) (Fig. 1B).

The allosteric effect(s) of FAs on Zn^2+^ binding to albumin provides a link between plasma Zn^2+^ homeostasis and FA metabolism. This is important because circulatory zinc handling can be affected when FA levels become elevated. Our recent work has shown that one result of this interaction between FAs and Zn^2+^ on albumin is an altered distribution of Zn^2+^ among plasma proteins (12). It may also promote Zn^2+^ uptake into endothelial cells (7). Notably, plasma FA levels are highly dynamic and are raised after fasting and under stress conditions (13, 14). However, chronically raised levels are associated with several disease states (e.g., obesity (15, 16), type 2 diabetes (4, 17), and cancer (18)). This aspect of lipid dyshomeostasis is proposed to alter circulatory zinc speciation, thus dysregulating both plasma and whole-body zinc dynamics. In turn, zinc dyshomeostasis likely contributes to vascular and metabolic pathologies associated with such disorders (4, 6). This is owed to the multiple effects of Zn^2+^ on hemostasis and energy metabolism mentioned above.

To better elucidate the role of crosstalk between FAs and Zn^2+^ in health and disease, the precise underlying molecular mechanism needs to be understood. While there are strong indications for the role of the FA2 site, its impact on the perturbation of Zn^2+^ binding to HSA has not been demonstrated experimentally. It is also unknown whether other sites also contribute to the observed allosteric effect. For example, the FA1 site is located within domain I, and FA6 and FA7 are within domain II and could also contribute to FA-responsive Zn^2+^ binding. The degree to which FAs impact Zn^2+^ binding at the secondary Zn^2+^ site is also unclear. Here, we used site-directed mutagenesis to examine how FA loading influences the binding of Zn^2+^ to recombinant albumin forms expressed in *Pichia pastoris* with targeted substitutions affecting both the primary and secondary Zn^2+^ sites as well as the FA2 site. Our study also utilized an innovative molecular dynamics (MD) simulation approach to investigate the interdomain dynamics of FA binding in the context of varying FA-site occupancy. This novel methodology combines steered molecular dynamics (SMD) and umbrella sampling MD (US MD), integrating multiple collective variables (CVs) to capture the conformational transitions associated with FA loading and unloading in HSA. We employed this approach to estimate the free energy associated with allosteric rearrangements sampled during the simulation, thus providing valuable insights into the mechanistic underpinnings of protein allostery. By integrating experimentally derived structural data with the dynamic information obtained from MD simulations, our study offers a comprehensive understanding of protein dynamics at the mechanistic and atomistic levels, particularly focusing on allosteric regulation. Importantly, the proposed MD simulation protocol can be readily adapted for investigating allosteric rearrangements in other proteins, highlighting its versatility and applicability in the field.

## Materials and Methods

### Recombinant albumin production and purification

Recombinant human albumin and mutant forms were expressed and purified as described previously (19). Genes were expressed using the EasySelect Pichia Expression kit (Thermo Fisher Scientific, Paisley, UK). The coding sequence of mature HSA (corresponding to residues 19–609 of the albumin preproprotein) was amplified and cloned into the pPICZαB plasmid in frame with the α-mating factor secretion signal sequence. Oligonucleotide-directed mutagenesis was used to prepare constructs encoding mutated albumin forms (H9A, H67A, H247A, and Y150F/R257A/S287A—the latter mutant referred to as FA2-KO) using the GENEART™ Site-Directed Mutagenesis System (Thermo Fisher Scientific, UK). Successful cloning was confirmed by DNA sequencing. After confirmation, plasmid DNA from resultant clones was extracted and transformed into X-33 *Pichia pastoris* competent cells.

Colonies expressing the proteins of interest were grown overnight in 15–50 ml of sterile buffered minimal glycerol medium containing histidine medium containing 100 mg/ml zeocin at 28°C with shaking at 200 rpm. This starter culture was added to fresh sterile buffered minimal glycerol medium containing histidine (350–500 ml) and further cultured overnight until reaching log phase growth (*A*_600_ = 2–6). Cells were harvested and resuspended in 2–4 L of medium. The culture was grown for 5 days, and protein expression induced through addition of 0.5% methanol every 24 h. Finally, the supernatant was removed following centrifugation and purification achieved by affinity chromatography using a HiTrap Blue HP column (Cytiva, Little Chalfont, UK), followed by size-exclusion chromatography using a HiLoad Superdex-75 column; Cytiva). All preparations were performed on an ÄKTA Purifier (Cytiva). The final protein purity, as assessed by sodium dodecyl sulfate-polyacrylamide gel electrophoresis, was >95%. In addition, protein sequence and intact mass were confirmed by mass spectrometry. Proteins were dialyzed into the required buffers prior to binding experiments.

### Defatting HSA and mutant samples

FAs were removed from commercial HSA (Sigma-Aldrich, UK) and Y150F/R257A/S287A mutant samples using charcoal as described in a previously reported procedure (20). A solution of HSA (100 mg/ml) was prepared with 1 ml of MilliQ water at 296 K, and 50 mg activated charcoal (Darco; Sigma-Aldrich, Poole, UK) was added. The pH of the solution was adjusted to 3.0 by adding 0.2 M HCl, and the mixture was left in a tube rotator for 2 h at 277 K. Charcoal was removed by centrifugation at 20,000 *g* for 30 min at 277 K, and the pH was adjusted to 7.4 using 0.2 M NaOH. A similar procedure was followed for the Y150F/R257A/S287A mutant sample (10 mg); subsequently, a 30 kDa molecular weight cut-off protein concentrator (Amicon Ultracel-30; Sigma-Aldrich) was used to concentrate the samples to 0.5 mM for the ^13^C NMR experiments.

### Isothermal titration calorimetry

Experiments were performed using a MicroCal VP-ITC (Malvern Panalytical, Malvern, UK). The FAs were prepared in either methanol or ethanol before being incubated with albumin in the reaction buffer for 2 h at 37°C (1% final alcohol concentration). Prior to the titration, all reactants were degassed at room temperature for 15 min. In the case of Zn^2+^-binding experiments, recombinant albumin forms and zinc solutions were prepared in a buffer containing 50 mM Tris (tris(hydroxymethyl)aminomethane), 140 mM NaCl, pH 7.4. Injections were carried out by titrating 1.5 mM ZnCl_2_ into 50 μM albumin in presence of 0 or 5 M equivalents (mol. eq.) of palmitate (C16; Sigma-Aldrich, UK; #P9767). The interaction of myristate (C14) with the recombinant albumins was also measured by isothermal titration calorimetry (ITC). In this case, HSA at 12.5 μM was loaded into the calorimeter cell. The injection syringe was loaded with 500 μM of sodium myristate (Sigma-Aldrich). Notably, all reactants for myristate binding to HSA were prepared in ultrapure water due to insufficient solubility of myristate in buffer. For 35 injections in total, each experiment was conducted at 25°C, with stirring speed at 307 rpm. The volume of the first injection was 2 μl over 4 s followed by 34 more injections of 8 μl over 16 s each with space period of 210 s and 150 s for zinc and myristate titrations, respectively, allowing complete equilibration between injections. Heats of dilution were accounted for with blank titrations performed by injecting Zn^2+^ or myristate solution, as appropriate, into reaction buffer and subtracting the averaged heat of dilution from the main experiments. Data fitting was performed using either Origin v7 (OriginLab, Northampton, MA) or AFFINImeter (Santiago de Compostela, Spain).

### Preparation of ^13^C-palmitate-albumin complexes for NMR experiments

The ^13^C NMR experiments were adapted from an approach developed by the Hamilton lab (21, 22, 23). A ^13^C-labeled palmitate stock solution (25 mM, 1 ml) was prepared by heating 500 μl of aqueous solution of 1-^13^C-palmitic acid (CK Isotopes, Newtown Unthank, UK) in a water bath at 328 K and adding 1 M KOH until the FA was completely dissolved. Before using for NMR sample preparation, palmitate stock solution was heated in a water bath at 328 K. 1:1 HSA-FA complexes were prepared at the following final concentrations: 0.5 mM HSA, 0.5 mM 1-^13^C-palmitate, 20 mM KCl, and 10% D_2_O. The pH of the solution was adjusted to 7.4 using 0.1 M KOH or HCl. Samples were incubated overnight at 310 K, and the pH was checked and readjusted, if necessary, prior to acquiring ^13^C-NMR data.

### ^13^C-NMR spectroscopy

All NMR experiments were performed in 3 mm NMR tubes. 1D ^1^H-decoupled (waltz64) ^13^C NMR spectra were acquired at 310 K on a Bruker Avance III HD 500 spectrometer (Bruker, Coventry, UK) equipped with a 5 mm double-resonance DCH 500 cryoprobe optimized for ^13^C sensitivity and operating at 125.77 MHz for ^13^C. The spectral width was 260 ppm, and a 90° pulse of 10 μs was used. Chemical shifts are reported relative to tetramethylsilane at 0 ppm.

### MD studies

We employed SMD and US MD techniques to study the energetics of the conformational change in HSA, transitioning from the open state (as observed via crystallography in the presence of bound FAs) (9) to the closed state (FA-free). Both MD simulation methods enhance sampling within the configuration space, particularly in regions where the molecular system's energy landscape impedes ergodicity (24). SMD was utilized to explore potential pathways in transition between the open and closed states of HSA and to identify distinct protein conformations which could be used as starting configurations in subsequent US calculations. These calculations allowed us to quantify the actual free energy changes associated with the conformational transition.

The initial geometry in the SMD simulations was based on the PDB file 1E7H, representing HSA crystallized in the presence of palmitate (9). Only palmitate molecules within FA1-FA6 sites were included, as the representation of the palmitate molecule in the FA7 site was poorly modeled. From this starting point, three structural variants in the open conformation were prepared: noFA (all palmitate molecules removed), 5FA-noFA2 (retaining 5 palmitate molecules, with those at FA2 and FA7 removed), and 6FA (including 6 palmitate molecules with the one at FA7 removed). The end point was chosen as chain A of the structure with PDB ID: 1BM0 (25), representing HSA without any bound ligands in the closed conformation.

We determined the CVs describing the conformational transition in our SMD calculations based on insights from the DynDom server's results (26). These results included both the axis and the angle range necessary for the rotation between distinct fragments of HSA. We translated these data into a dihedral angle restraint (D1 in Supplemental Table S1) and used it in an SMD simulation to verify the overall completeness of the modeled transition. Our criteria for adequate modeling involved ensuring minimal divergence between the SMD-derived and the reference closed conformation of HSA domain I (PDB ID: 1BM0) (25). We considered the transition well-represented when the root mean square deviation calculated for the peptide backbone heavy atoms was below 0.25 nm. In our investigation, we meticulously examined the differences between the modeled and reference conformations of HSA domain I fragments contributing significantly to the root mean square deviation and iteratively expanded our set of CVs as necessary. The set of eight CVs reported here comprises four dihedral angle (D1–D4 in Supplemental Table S1), one planar angle (P1), and three distance (R1–R3) restraints. These variables collectively enabled us to effectively replicate the transition between open and closed HSA structures (Supplemental Fig. S1). Further details concerning settings of pulling rates and force constants for CVs are given in Supplemental Table S1.

We selected 28–39 distinct HSA conformations (see Supplemental Table S2) for our US simulations, representing various stages along the conformational transition pathway identified in the final SMD. We aimed to ensure adequate overlap between consecutive regions of the sampled conformational space governed by different CVs. The results of US calculations were combined with the weighted histogram analysis method (27) to determine the free energy as a function of distinct CVs that described the conformational transition.

We conducted MD simulations using Gromacs 2022 (27, 28). CHARMM 36 force field parameters (29, 30) were assigned to HSA and palmitate with CHARMM-GUI (31). Ionization states of HSA amino acid residues at pH 7.4 were determined using the PropKa server (32). For water, we used standard TIP3P parameters (33). We neutralized the system employing 150 mM NaCl. Further details of the simulated molecular systems are given in Supplemental Table S3. All MD simulations were carried out at 37°C and 1 bar. Temperature and pressure were controlled by Nose-Hoover thermostat (34) and Parrinello-Rahman barostat (35), respectively. The MD simulations were conducted employing periodic boundary conditions, utilizing a rhombic dodecahedron as the geometry for the simulation box. The integration time step was set to 2 fs. Simulations in the SMD and in individual US runs spanned 5 ns and 10 ns, respectively. Combining these simulations across all systems totaled a duration of 1 μs.

## Results

### Effects of FAs on Zn^2+^ binding to site A- and site B-targeted HSA mutants

**Fig. 2:**
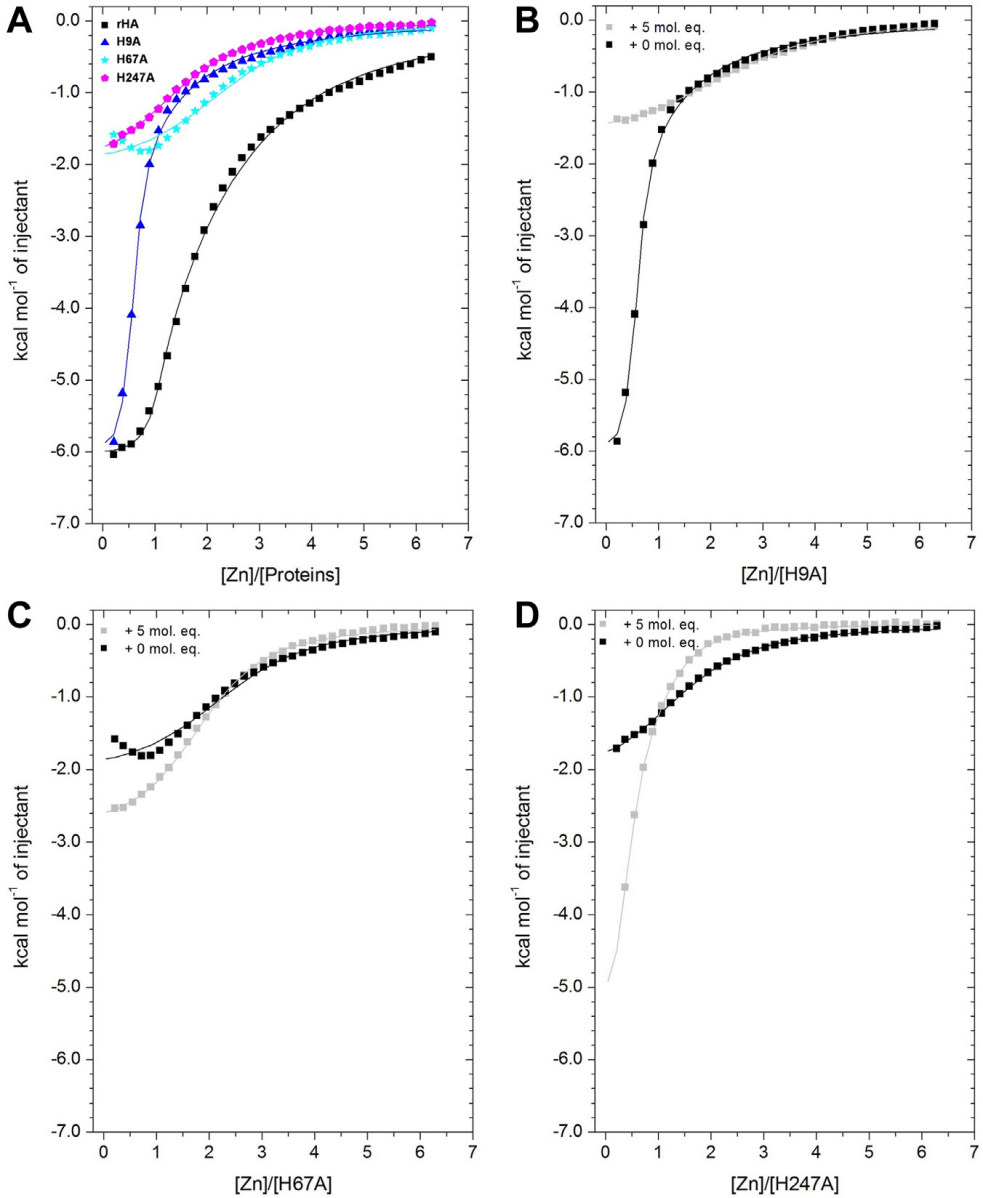
Zn^2+^ binding to rHA, H9A, H67A, and H247A proteins in the presence and absence of 5 mol. eq. of palmitate. ITC spectra of (A) Zn^2+^ binding to rHA, H9A, H67A, and H247A mutant proteins. B: Zn^2+^ binding to H9A with 0 and 5 mol. eq. of palmitate bound. C: Zn^2+^ binding to H67A with 0 and 5 mol. eq. of palmitate bound. D: Zn^2+^ binding to H247A with 0 and 5 mol. eq. of palmitate bound.

Using ITC, we have previously demonstrated that the binding of various plasma FAs including myristate (14:0) and palmitate (16:0) strongly reduce Zn^2+^ binding to HSA (4). We adopted the same approach to study the effect of bound palmitate on Zn^2+^ binding to albumin mutants targeting His residues in site A (H67A and H247A) and site B (H9A). Firstly, the effect of the three mutations was established in the absence of FAs. In accordance with previous work (4), Zn^2+^ titration data obtained for recombinant HSA (rHA) fitted well with a two-sets-of-sites model where both N1 and N2 were fixed to 1 (Fig. 2A; Supplemental Table S4). Data could also be fitted with a similar model where N2 was fixed to 2 (albeit with a higher χ^2^ value). Although site B forms a secondary site, multiple weaker sites exist (8) and may contribute to the ITC data. A reduction in Zn^2+^ binding by all three mutants is evident from the data plotted in Fig. 2A. Data fitting to a two-sets-of-sites model with K_ITC__1_ and ΔH1 fixed (based upon obtained values for rHA data) and other parameters allowed to vary gave N1 = 0 for the H67A and H247A mutants. As expected, this indicates that both His67 and His247 play an indispensable role in Zn^2+^ binding to site A. However, N2 increased to 2.47 and 1.66 in the H67A and H247A mutants, respectively. This may indicate that single mutations in site A may give rise to the formation of a weaker Zn^2+^-binding site that involves the remaining available ligands, with an affinity similar to that of site B.

Zn^2+^ binding to the H9A mutant was also examined. While in this case, initial binding remained strong, the binding curve was shifted toward lower stoichiometries relative to rHA. Together with inspection of the fitting results, this suggests that removal of His9 has a minor impact on the highest-affinity site but decreases Zn^2+^ binding to the secondary site of HSA, consistent with His9 being a site B ligand. A two-sets-of-sites model was used to fit the corresponding data, with K_ITC1_, K_ITC__2_, ΔH1, and ΔH2 fixed (based on the obtained rHA data), and the N parameters allowed to vary. This yielded a reasonable fit, with N1 and N2 decreasing to 0.51 and 0.34, respectively (Supplemental Table S4). As previously observed (4), the equilibria for sites A and B overlap and therefore often cannot be deconvoluted. Nonetheless, the loss of approximately one binding site in the H9A mutant strongly supports the hypothesis that this residue forms part of site B.

Next, the effect of 5 mol. eq. palmitate bound to the H9A, H67A, and H247A mutants was examined (Fig. 2B–D). For the H9A mutant (site B compromised), the presence of palmitate led to a strong reduction in Zn^2+^ binding at the remaining high-affinity site, consistent with the FA greatly diminishing the availability of site A. Indeed, data fitting using a two-sets-of-sites model supported a reduction in site A stoichiometry (from 0.51 to zero). The residual binding observed is likely predominantly due to weak (tertiary) sites (8). Somewhat unexpectedly, the presence of palmitate enhanced binding to the two site A mutants (H67A and H247A). In the case of the H67A mutant, the presence of palmitate abolished the endothermic component of the thermogram. The K_ITC2_ resulting from fitting was also slightly increased. For the H247A mutant, K_ITC2_ increased by a factor of almost 2.

### Targeting FA binding at the FA2 site using mutagenesis

**Fig. 3:**
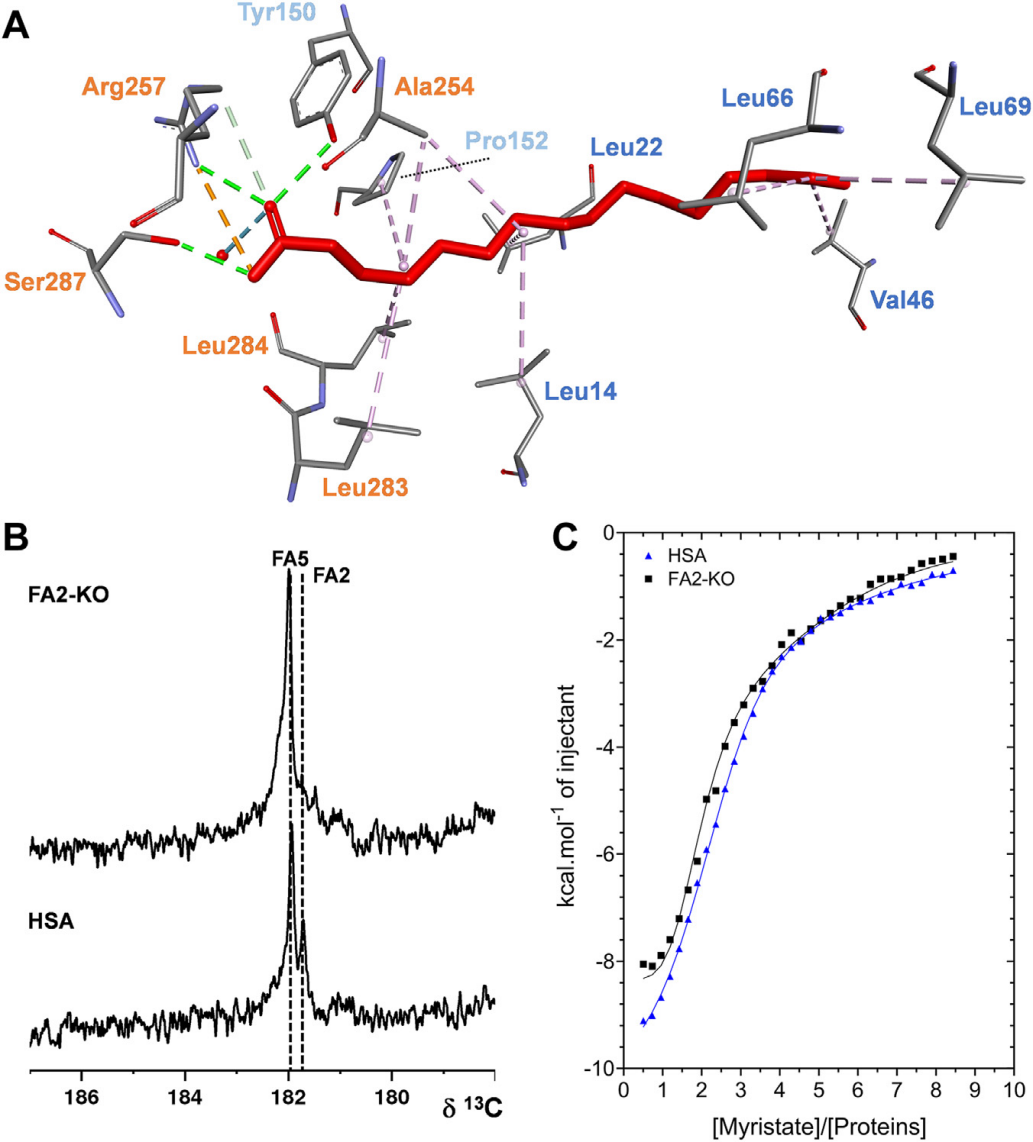
FA binding to HSA and FA2-KO mutant proteins. A: Structure of palmitate bound at the FA2 site (based on coordinates from PDB: 1E7H) (9) showing interactions with residues in subdomains IA (blue), IB (light blue), and IIA (orange). Residues Tyr150, Arg257, and Ser287 provide H-bonds stabilizing the carboxylate group of the bound FA. B: ^13^C-NMR spectra of 1-[^13^C]-palmitate binding to native HSA (commercially obtained from plasma) and FA2-KO mutant. In each case, spectra were recorded at a 1:1 albumin:FA ratio. Binding to HSA gave rise to two distinct peaks at 181.7 and 181.9, corresponding to sites FA2 and FA5, respectively (24, 25). The peak corresponding to FA binding at FA2 was absent from the FA2-KO protein spectra. C: ITC analysis of myristate binding to native HSA and the FA2-KO mutant protein. Myristate (500 μM) was titrated into a solution containing 12.5 μM protein. Titrations were carried out in H_2_O due to insolubility of myristate in buffered solutions.

To examine the impact of FA binding at the FA2 site of albumin on Zn^2+^ binding, a triple mutant form of the protein (referred to as FA2-KO) was generated, possessing substitutions at residues Tyr150 (to Phe), Arg257 (to Ala), and Ser287 (to Ala). These residues were chosen as their sidechains form essential interactions with the carboxylate group of FA molecules bound at the FA2 site (Fig. 3A) (9, 23). Substituting these residues for others that lack the required groups to form these contacts was thus expected to greatly perturb FA binding at this site. Binding of 1-[^13^C]-palmitate to wild type and FA2-KO was probed using ^13^C-NMR. Previous work by Simard *et al.* revealed that 1-[^13^C]-palmitate binds with highest affinity at sites FA2 and FA5 (21, 22). For each of the proteins, ^13^C-NMR spectra of 1-[^13^C]-palmitate complexes (at a 1:1 protein:FA ratio) were obtained (Fig. 3B). Binding to HSA gave rise to two distinct peaks at 181.7 and 181.9 ppm, corresponding to FA binding at sites FA2 and FA5, respectively (21, 22). The peak corresponding to 1-[^13^C]-palmitate binding at FA2 in HSA (at 181.7 ppm) was absent from the FA2-KO mutant spectra, consistent with the mutations removing binding at this site. The binding of myristate to wild-type and FA2-KO albumin was also examined using ITC. This experiment was carried out essentially as previously described for bovine serum albumin (10). Myristate bound readily and with exothermic energetics to wild-type and FA2-KO albumin, with the data for the mutant indicating a reduction in stoichiometry (Fig. 3C). Several fitting models were explored, based upon the knowledge that HSA possesses seven FA sites (2–3 high affinity sites and several lower affinity sites) (9, 36). These are summarized in Supplemental Table S5. A three-sets-of-sites model (with stoichiometries of sites fixed to N1 = 2.0, N2 = 1.0, and N3 = 4.0 for highest, medium and lowest affinity sites, respectively) fitted the HSA data well. Simpler two-sets-of-sites models failed to result in reasonable fits. The obtained K_ITC_ values for the wild-type protein were K_ITC1_ = 1.76×10^6^ M^−1^, K_ITC2_ = 3.91×10^5^ M^−1^, and K_ITC3_ = 3.11×10^4^ M^−1^, respectively. For the FA2-KO mutant, a similar three-sets-of-sites fitting strategy was adopted, but the stoichiometry of N1 (the highest affinity set-of-sites) was changed from 2.0 to 1.0. During fitting, the K_ITC_ and ΔH values for all sites were allowed to vary. The data could be successfully fitted using this model. The obtained K_ITC_ values for the FA2-KO protein were K_ITC1_ = 1.60×10^7^ M^−1^, K_ITC2_ = 1.23×10^6^ M^−1^, and K_ITC3_ = 8.13×10^4^ M^−1^, respectively. Overall, both NMR and ITC data are consistent with the amino acid substitutions removing the ability of FA2 to bind FAs under the conditions examined.

### FA2 site mutations protect HSA-Zn^2+^ binding against FA effects

**Fig. 4:**
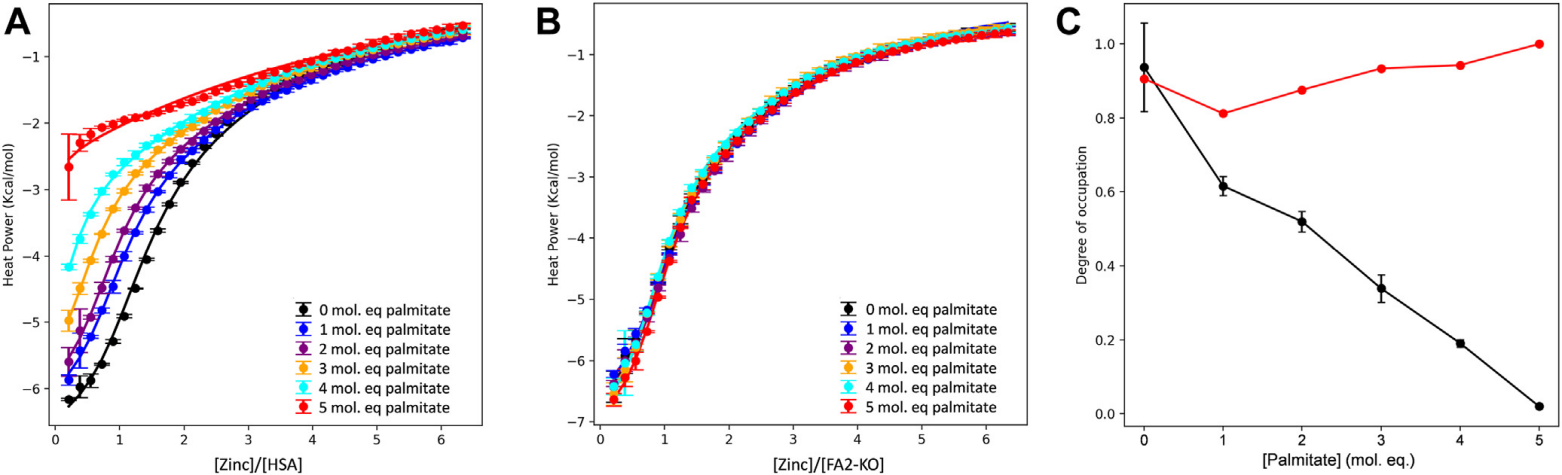
Zn^2+^ binding to HSA and FA2-KO proteins loaded with palmitate. Global fitting of Zn^2+^ binding to HSA and FA2-KO proteins loaded with palmitate. A: ITC spectra showing Zn^2+^ binding to rHA loaded with 0–5 mol. eq. of palmitate (palm). B: Global fitting of Zn^2+^ binding to the FA2-KO mutant albumin loaded with 0–5 mol. eq. of palmitate. C: Resultant estimated occupation of site A in both HSA (black) and FA2-KO (red) proteins.

To determine the specific role that FA binding at the FA2 site plays in the inhibition of Zn^2+^ binding, we used ITC to examine Zn^2+^ binding to HSA and FA2-KO albumins loaded with different concentrations of palmitate. Note that Zn^2+^ binding to HSA and recombinant wild-type albumin, as assessed by ITC, gave comparable results. As observed previously (4), Zn^2+^ binding to HSA was reduced in the presence of increasing palmitate (0–5 mol. eq. relative to albumin) in a concentration-dependent manner (Fig. 4A). In contrast, the ITC plots of Fig. 4B immediately highlight that the FA2-KO mutant does not respond to the presence of FA in the same manner as the wild type.

After an initial analysis of the ITC data for wild-type and FA2-KO mutant albumins using the two-sets-of-sites approach described above (Supplemental Fig. S2 and Supplemental Table S6), the viability of a global fitting approach using AFFINImeter software was explored (37). Guided by previously employed models for fitting single isotherms, a two-sets-of-sites model was used to fit all 12 isotherms for the two albumins simultaneously. The model was based on both proteins having one set of one high-affinity site and another set of two weaker binding sites. All thermodynamic parameters (K_ITC_ and ΔH values) were shared across the isotherms; and only the degree of occupation (Θ, analogous to N1 in single-isotherm fits) of the high-affinity binding site was set as the fitting variable parameter. The lines shown in Fig. 4A, B correspond to the fitted model, with parameters recorded in Supplemental Table S7. The resulting binding constants (K_ITC1_ = (2.9 ± 0.1) × 10^5^ and K_ITC2_ = (2.5 ± 0.2) × 10^4^ are in good agreement with those obtained from single isotherm fits for wild-type HSA and rHA (Supplemental Tables S4 and S6) (4, 8). The fact that this global fitting approach is viable may suggest that the underlying hypothesis is reasonable. Crucially, the degree of occupation of the high-affinity binding site (Fig. 4C) significantly decreased in response to increasing palmitate concentration for wild-type HSA but remained near 1 for the FA2-KO mutant at all palmitate concentrations.

### MD simulations

**Fig. 5:**
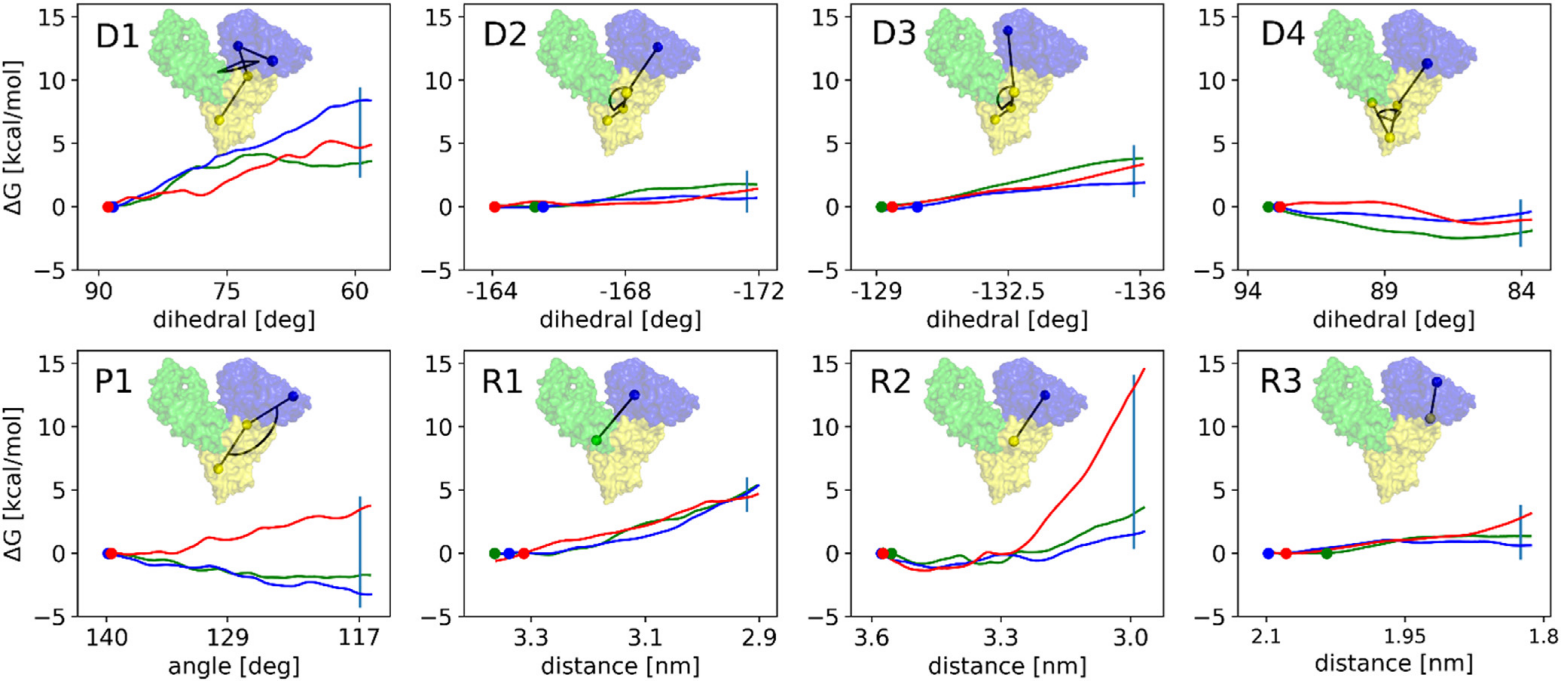
The free energy (ΔG) profiles associated with individual collective variables (CVs) during conformational rearrangements for each model system. The individual data are indicated as follows: noFA (green lines), 5FA-noFA2 (blue lines), and 6FA (red lines). The labels for the CVs are given in the top left corners of the plots. HSA is depicted with its distinct domains color-coded as follows: domain I (dark blue), domain II (yellow), and domain III (light green). The balls and sticks displayed on the plots symbolize specific CVs, where each ball corresponds to the center of mass of atom groups defining these variables. Vertical light blue lines on the plots indicate the target CV values representing the closed conformation (PDB ID: 1BM0).

Comparisons between experimentally determined structures of HSA with and without bound FAs offer detailed insight into the distinct conformational states of the protein. However, they do not capture the dynamic alterations in the protein's structure upon FA binding. To address this, we developed a combined approach using SMD and US MD simulations. Our aim was to explore the changes in free energy (ΔG) associated with the transition between two distinct structural states of HSA: an 'open' and a 'closed' conformation. The ‘open’ conformation denotes the state typically observed when all FA molecules are bound to HSA, while the ‘closed’ conformation corresponds to that of ligand-free HSA. The impact of bound FAs was explored by comparing the MD trajectories from the open to the closed form in the absence and presence of palmitate. Initially, we attempted to characterize the conformational transition using CVs obtained from the DynDom server (26), but this proved insufficient. Subsequently, we devised seven additional CVs. Together, these eight CVs (Fig. 5) provided a more comprehensive framework, enabling us to thoroughly sample the transition pathway (Supplemental Fig. S1).

Three model systems were subjected to MD simulations: noFA (FA-free albumin), 6FA (all well-defined FA-binding sites occupied), and 5FA-noFA2 (as 6FA but empty FA2). Hence, comparison between 6FA and 5FA-noFA2 should allow to assess the effect of FA bound in site FA2. It needs to be noted that the MD simulation time covered in each US window is inherently too short to allow FA molecules to dissociate from HSA-binding pockets. This allows estimating ΔG differences for "frozen" intermediate states along the transition pathway. The ΔG profiles for the three protein model systems along D2, D3, D4, R1, and R3 CVs exhibit considerable similarity, while the profiles for D1, P1, and R2 CVs differ significantly. Specifically, the dihedral angle D1 governs the relative rearrangement between domain I and domain II, while the planar angle P1 plays a crucial role in effectively closing FA-binding site 2, spanning the interface between these domains. Meanwhile, the distance R2 facilitates the completion of these conformational changes by decreasing the separation between domains I and II.

**Fig. 6:**
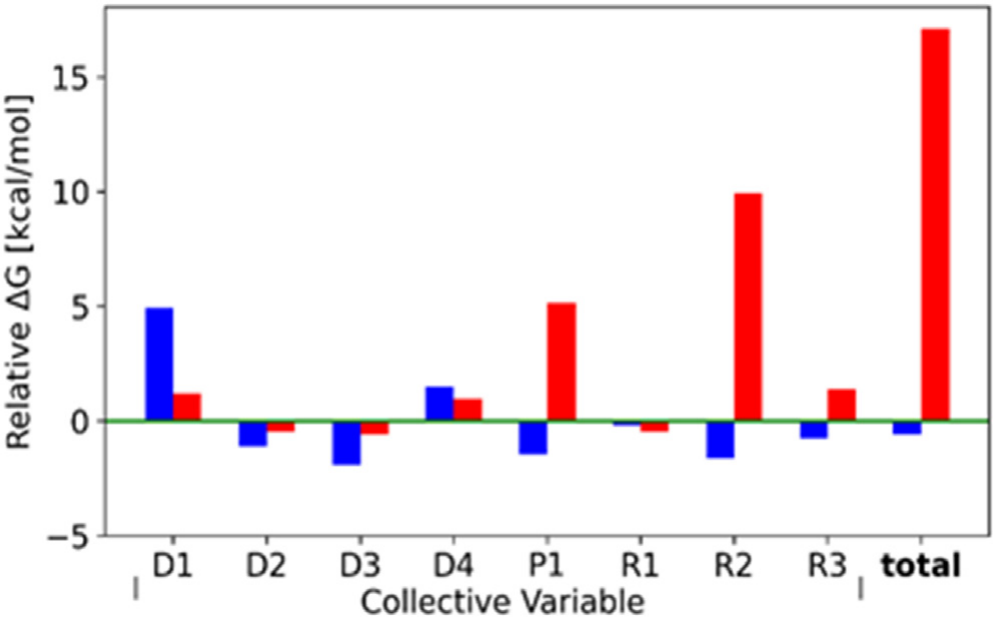
Free energy (ΔG) changes for collective variables (CVs) in each model system. Histograms of calculated ΔG values for 5FA-noFA2 (blue bars) and FA6 (red bars) models relative to the noFA (green base line) model system for each of eight CVs. The free energy changes were read at CV target values corresponding to the closed conformation of HSA (PDB ID: 1BM0). Total is the sum of the respective ΔG values.

In evaluating how different CVs contribute to the overall free energy during HSA's conformational transition, we extracted the specific ΔG values from each of the eight plots at the CV values corresponding to the protein's final closed state (marked by vertical blue lines in Fig. 5). By using the FA-free system (noFA) as the point of reference, we calculated the relative ΔG values for 5FA-noFA2 and 6FA. Figure 6 shows these results alongside the cumulative relative ΔG change, which combines the contributions from individual CVs. Notably, the data presented in Figs. 5 and 6 highlight that the energetics in the 6FA system are distinct from the two models lacking an occupied FA2: the trajectories show a rapid rise in ΔG when R2 decreases below 3.25 nm, coinciding with a moderate increase in ΔG when P1 drops below 134°; this is also reflected in the high positive values for P1 and R2 in Fig. 6. However, it is imperative to acknowledge that the additional CVs play a crucial role in facilitating the conformational change (Supplemental Fig. S1). Nevertheless, their impact on the overall change in ΔG during the transition remained relatively unaffected by the presence of FA in FA2.

## Discussion

Mutation of key site A (His67 and His247) and site B (His9) residues led to a reduction in Zn^2+^ binding in the ITC experiments. Zn^2+^ binding at site A is complex, and structural work on ESA has shown that in the presence of high concentrations of Zn^2+^, the His246 (His247 in HSA) sidechain can rotate away from His67 and Asp248 (Asp249 in ESA) and instead accommodate an additional Zn^2+^ ion, with the rest of the coordination sphere provided by water molecules, leaving His67 and Asp248 as the only residues coordinated to the other Zn^2+^ ion. This was observed in ESA crystals soaked in a high concentration Zn^2+^ solution (∼30 mM) (8). Although this behavior has not been confirmed to occur in HSA (or indeed in solution), these residues are completely conserved between ESA and HSA. It is unlikely that the extra site involving His247 would manifest itself in the ITC data, but it is possible that the 2-residue site (His67 and Asp249) may be observable as a relatively weak site, which may explain why when examining Zn^2+^ binding to the H247A mutant, N2 was >1 (*ca.* 1.66) (Supplemental Table S4).

The isotherm for the H67A mutant—like those obtained previously for this mutant (8)—featured an endothermic component on addition of up to ∼1.5 mol. eq. of ZnCl_2_. This could indicate that initial binding to at least one of the remaining sites is impeded in some way, perhaps by subtle structural changes induced by the absence of this imidazole sidechain. In the unliganded HSA structure (PDB: 1BM0) (25), the imidazole groups of both His67 and His247 form interdomain H-bonds (in the case of His67 an H-bond to the backbone carbonyl oxygen of Gly248), providing stability at the domain IA-IIA interface (Supplemental Fig. S3). Although we have previously shown by ^1^H-NMR that these mutants form ordered structures like the native protein (19), the H67A and H247A mutations would lead to removal of these respective interactions and trigger some localized destabilization of the domain interface. As there are few H-bonds at this domain interface, removing one may be quite impactful. This may disturb the preorganization of site B, which is also located at the domain IA-IIA interface. The requirement for additional structural changes to form site B in the H67A mutant may be the origin of the endothermic component. Zn^2+^ binding to the H9A mutant was also examined. Removal of His9 had a minor impact on the highest-affinity site but decreased Zn^2+^ binding to the secondary site of HSA, which is consistent with His9 being a site B ligand.

Previously, we examined how Zn^2+^ displaced from albumin by FAs might bind to and activate another zinc-binding protein, histidine-rich glycoprotein (HRG)—a Zn^2+^-responsive adaptor protein also found in plasma that regulates coagulation, angiogenesis, and immune functioning (38). Speciation modeling was performed incorporating typical plasma concentrations as well as the affinities and stoichiometries of sites A and B on albumin and the Zn sites on HRG, based on data obtained in the presence and absence of 5 mol. eq. of myristate. The model incorporated the assumption that FA binding does not affect site B, which is largely supported by the current study. At a typical HSA plasma concentration of 620 μM, site B accounted for 5%–10% of zinc species in the absence and 60%–70% in the presence of myristate. The amount of Zn^2+^ bound to HRG increased about 10-fold, with unbound Zn^2+^ also increasing from 0.5% to roughly 5%–10%. If we assume that HRG in this model serves as a proxy for other zinc-binding proteins, the data suggest that while site B has the potential to pick up some Zn^2+^ when site A is unavailable, it is, despite its high concentration, unable to fully compensate for a compromised site A.

The effect of 5 mol. eq. palmitate bound to the H9A, H67A, and H247A mutants was also examined. Palmitate led to a strong reduction in Zn^2+^ binding at the remaining (high-affinity) site of H9A, as supported by the data fitting. Unexpectedly, the presence of palmitate enhanced binding to H67A and H247A. We suggest that in both cases, these small increases may result from FA-induced stabilization of the domain interface, essentially reversing the hypothesized destabilization in the non-FA-loaded H67A mutant. It has been known since the 1940s that FAs can stabilize albumin against heat, urea, and guanidine denaturation (39, 40). As the FA2 site is composed of two pockets, bound FA can potentially act like a “pin” and thus stabilize the domain IA-IIA interface. It is also possible that FA binding at FA2 promotes the formation of new binding sites. Examination of a palmitate-bound HSA structure (PDB: 1E7H) (9) reveals that the positions of His67 and Asp249 sidechains are located within 3.0 Å of each other, and the sidechain of Asn99 is also nearby and could provide an additional ligand for Zn^2+^ coordination (Supplemental Fig. S4). The sidechain of Glu252 is also within ∼5 Å and could potentially rotate to coordinate Zn^2+^.

Interestingly, from a phenomenological standpoint, Zn^2+^ binding to HSA has been shown to impact on FA binding. We previously explored the effect of Zn^2+^ on myristate binding to albumin (10), where an equimolar amount of Zn^2+^ did not change stoichiometry of myristate binding compared to bovine serum albumin (12.5 μM) in the absence of Zn^2+^ but did reduce the overall affinity. However, this relationship is likely to be of little physiological importance, as less than 1 in 30 albumin molecules carry a Zn^2+^ ion under normal conditions; thus, the relatively small changes of zinc concentrations observed physiologically are not expected to significantly impact FA transport.

FA binding at the FA2 site of HSA was targeted using a triple mutation approach (to generate the FA2-KO form of HSA). NMR and ITC experiments confirmed that FA binding at FA2 was diminished by the mutations. Furthermore, unlike with wild-type HSA, palmitate loading of FA2-KO had no effect on Zn^2+^ binding. This suggests that FA binding at the FA2 site is solely responsible for the allosteric inhibition of Zn^2+^ binding and that the binding of FA molecules at other sites on albumin do not significantly contribute to this effect.

We believe that this is the first study to generate an engineered albumin that lacks a specific “FA binding site”. However, it is noteworthy that Sudlow sites I and II on HSA, which bind and transport small organic molecules such as drugs, have previously been characterized by site-directed mutagenesis (41). In this study, it was shown that substitutions at these sites impact upon the binding of various FAs. The abolition of the “FA/Zn^2+^-switch” in the FA2-KO mutant may render this mutant form useful for future functional studies aimed at understanding the physiological importance of the FA2 site or indeed, the effects of its occupation upon plasma Zn^2+^ handling. Several studies, including meta-analyses, have examined total plasma/serum zinc concentrations in type 2 diabetes patients, with either high (42) or low (43, 44) serum zinc concentrations being associated with type 2 diabetes. We recently examined plasma metal levels in diabetes patients and found no difference in total zinc in plasma from type 2 diabetes patients compared to age-matched controls (45). Thus, the relationship between total plasma zinc concentration and incidence of type 2 diabetes is not clear. Indeed, the zinc status of an individual is not reflected well by the plasma zinc concentration (46). What is however likely of greater importance than the total zinc level in serum/plasma is how that zinc is handled and its resultant “speciation”—which is dominated by albumin. If FA levels are sufficiently high as to compromise normal zinc handling by albumin, more zinc will bind to other proteins or be taken up by cells, as we have observed before (7, 12). As well as having effects on cellular zinc flux, which may influence metabolic processes (47), albumin-Zn^2+^ interactions have been reported to be important for regulating insulin signaling (5). Insulin is released from the pancreas as an inactive hexameric molecule complexed with two Zn^2+^ ions (5, 6). Increased FA binding to HSA, due to elevated FA levels, may contribute to altered insulin signaling dynamics in type 2 diabetes by reducing the capacity of HSA to compete with insulin for Zn^2+^ binding (6). This may reduce the proportion of active monomeric or dimeric insulin.

Incidence of type 2 diabetes is associated with the formation of denser, less porous blood clots than in healthy individuals, which contribute to the increase in CVD risk in such patients (48). We have shown using a turbidimetric fibrin clot assay that the addition of Zn^2+^ to plasma leads to an increase in maximum turbidity (reflecting the size and/or density of the clot formed) upon addition of thrombin and Ca^2+^, while FA concentrations correlate with maximum turbidity when the same assay is performed on plasma taken from type 2 diabetes patients and controls (4). Fibrin clot turbidity is known to reflect adverse clinical outcomes (49). This suggests that the FA/Zn^2+^ switch may contribute to the increased risk of thrombotic complications observed in type 2 diabetes (50).

SMD and US MD simulations were employed to explore the changes in free energy (ΔG) associated with the transition between the 'open' FA-bound and 'closed' apo-structural states of HSA for three systems (no FA, 5FA-noFA2, and 6FA). The data presented highlight that the energetics in the 6FA system are distinct from the models lacking an occupied FA2, suggesting that FA binding at FA2 stabilizes the open conformation. Collectively, these observations shed light on the mechanism through which FA binding at the FA2 site sterically impedes the adoption of the closed conformation necessary to accommodate a Zn^2+^ ion at site A (His67, His247, and Asp249): When R2 reaches approximately 3.25 nm, the FA in FA2 becomes constrained between the terminal groups of R2 (Supplemental Table S1), causing structural rearrangements within HSA to lag behind the imposed movement along R2. Consequently, this results in an almost linear rise in ΔG. The same rationale can be applied to the increase in relative ΔG for P1. Although this CV is defined by different atom groups within domains I and II of HSA (Supplemental Table S1), decrease in P1 results in narrowing of the space within the FA2-binding site, which eventually becomes too small to accommodate the FA molecule.

## Conclusions

In summary, the studies of the allosteric mechanism of FA/Zn^2+^-binding switch on human albumin suggest that Zn^2+^ binding to site A is more affected by FA binding than that to site B. Our data also show that FA binding at the FA2 site can be targeted using site-directed mutagenesis. Furthermore, both experimental (ITC) and theoretical (MD simulations) approaches have shown that FA2 is solely responsible for the FA/Zn-switch on HSA. Targeting FA2-specific FA interactions will be helpful for further functional studies focused on exploring the interplay between circulating FA levels and plasma Zn^2+^ speciation in health and disease. Collectively, this work provides new mechanistic understanding of how FAs regulate plasma Zn^2+^ handling through their interaction with HSA. These findings may be exploited to better understand a variety of Zn^2+^-regulated circulatory processes, including cellular uptake, hemostasis, and regulation of insulin dynamics.

## Data availability

The research data supporting this publication can be accessed at https://doi.org/10.17630/ae9d50a4-e164-454a-86ee-330d654214b1 (51).

## Supplemental data

This article contains supplemental data (4, 8, 9, 25).

## Conflict of interest

J. S. is a co-founder and CEO at AFFINImeter. All other authors declare that they have no conflicts of interest with the contents of this article.
