# Supplementary material for: Targeted removal of the FA2 site on human albumin prevents fatty acid–mediated inhibition of Zn2+ binding
Source: J Lipid Res. 2024 May 14;65(6):100560. doi: 10.1016/j.jlr.2024.100560 (PMC11179626; doi:10.1016/j.jlr.2024.100560)
Supplement: Supplemental Data [file mmc1.docx]

**Targeted removal of the FA2 site on human albumin prevents fatty acid-mediated inhibition of Zn^2+^-binding**

Dongmei Wu, Stephen J. Hierons, Sirilata Polepalli, Michal Gucwa, Remi Fritzen, Michal Markiewicz, Juan Sabín, Wladek Minor, Krzysztof Murzyn, Claudia. A. Blindauer and Alan J. Stewart

**Supplementary Information**

**Table S1. Collective variables (CVs) used in the steered molecular dynamics simulations.**

**Figure S1. Results of steered molecular dynamics simulations conducted for noFA, 5FA-noFA2, and 6FA systems.**

**Table S2. Collective variables ranges sampled in umbrella sampling MD simulations for noFA (29 US windows), 5FA-noFA2 (39), and 6FA (28) systems.**

**Table S3. Details on molecular composition and dimensions of model systems in the MD simulations.**

**Table S4. Fitting approach used for ITC studies examining Zn^2+^ binding to rHA and H9A, H67A and H247 mutant albumins and resulting data.**

**Table S5.** **Fitting approach used for ITC studies examining myristate binding to HSA and Y150F/R257A/S287A albumin and resulting data.**

**Table S6. Fitting approach used for ITC studies examining Zn^2+^ binding to rHA and Y150F/R257A/S287A albumin and resulting data.**

**Figure S2. Zn^2+^-binding to HSA and FA2-KO proteins loaded with palmitate.**

**Table S7. Resulting measured parameters for the global fitting of Zn^2+^-binding to HSA and FA2-KO proteins loaded with palmitate (shown in Figure S3).**

**Figure S3. Domain IA-IIA interface at site A of unliganded HSA.**

**Figure S4. Domain IA-IIA interface at sites A of HSA with palmitate bound.**

**Table S1. Collective variables (CVs) employed in the steered molecular dynamics simulations.** The variables are defined based on the centre of mass (CoM) for distinct groups of peptide backbone atoms (N, C, and C-alpha) within specified amino acid residue ranges. The table includes details such as pulling force constants (K), pulling rates, and boundary values (Start, End) for each CV, corresponding to the open (PDB ID: 1E7H) and closed (PDB ID: 1BM0) conformations of HSA.

| **CV Label** | **CV Type** | **CoM** | **Start** | **End** | **K** | **Rate** |
| --- | --- | --- | --- | --- | --- | --- |
| D1 | Dihedral | A: 85-105 B: 158-162+ 182-186 C: 289-293 D: 340-383 | 88.7° | 59.4° | 3000 [kJ mol^-1^ rad^-2^] | -8.08164  [deg ns^-1^] |
| D2 | Dihedral | A: 17-20 B: 204-207 C: 331-335 D: 340-383 | -164.3° | -171.6° | 3000  [kJ mol^-1^ rad^-2^] | -2.29152  [deg ns^-1^] |
| D3 | Dihedral | A: 164-180 B: 204-207 C: 331-335 D: 340-383 | -128.8° | -135.8° | 5000 [kJ mol^-1^ rad^-2^] | -4.015264  [deg ns^-1^] |
| D4 | Dihedral | A: 101-105 B: 333-338 C: 358-362 D: 380-383 | 93.066° | 84.042° | 3000 [kJ mol^-1^ rad^-2^] | -3.320976  [deg ns^-1^] |
| P1 | Angle | A: 50-55 B: 289-293 C: 340-383 | 139.5° | 117.0° | 3000 [kJ mol^-1^ nm^-2^] | -7.114536  [deg ns^-1^] |
| R1 | Distance | A: 142-145 B: 483-488 | 3.35 nm | 2.92 nm | 3000 [kJ mol^-1^ nm^-2^] | -0.1455464  [nm ns^-1^] |
| R2 | Distance | A: 17-20 B: 204-207 | 3.55 nm | 2.99 nm | 3000 [kJ mol^-1^ nm^-2^] | -0.1890672  [nm ns^-1^] |
| R3 | Distance | A: 40-43 B: 250-253 | 2.08 nm | 1.83 nm | 5000 [kJ mol^-1^ nm^-2^] | -0.0872976  [nm ns^-1^] |

**Figure S1. Results of steered molecular dynamics simulations conducted for noFA, 5FA-noFA2, and 6FA systems.** Eight collective variables were employed to induce conformational changes from the open (start at time 0) to closed state (around 3 ns). The transition of the protein in SMD was described by comparing MD conformations with reference structures: 1E7H (open state, orange) and PDB 1BM0 (closed state, purple-IA, dark purple-IB). The first step for conducting comparisons was the fit of domain II to the reference structure. Subsequently, using this fit, the root mean square deviation (RMSD) of the backbones of domains IA (first row) and IB (second row) was calculated. The third row illustrates the fit of conformations selected with a black dot in the first two rows to the reference structure PDB 1BM0. The red shading represents the fitting region, while purple and dark purple indicate regions for domain IA (16-107) and IB (119-196), respectively, along which the RMSD presented here was calculated. The remaining residues in reference structures 1BM0 are coloured pink, and the rest of the structure from SMDs is represented in grey.


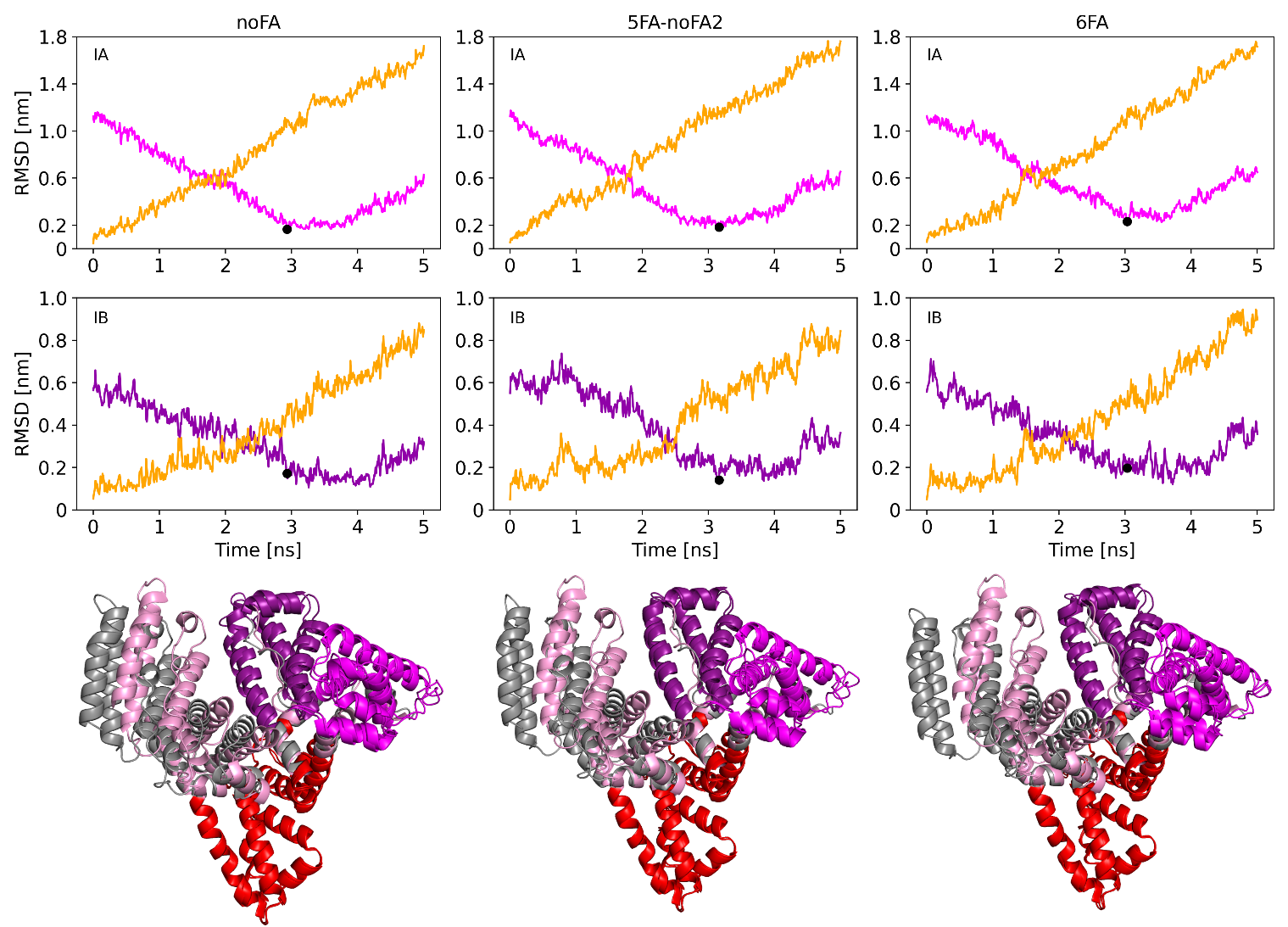


**Table S2. Collective variables ranges sampled in umbrella sampling MD simulations for noFA (29 US windows), 5FA-noFA2 (39), and 6FA (28) systems.** For each CV, the top and bottom values correspond to open and closed states of HSA, respectively. The biasing force constants ranged from 6000 to 12000 (in kJ mol^-1^ nm^-2^ or kJ mol^-1^ rad^-2^).

| **Model System** | **D1**  **[deg]** | **D2** **[deg]** | **D3** **[deg]** | **D4** **[deg]** | **P1** **[deg]** | **R1** **[nm]** | **R2** **[nm]** | **R3** **[nm]** |
| --- | --- | --- | --- | --- | --- | --- | --- | --- |
| noFA | 88  57 | -164  -175 | -129  -140 | 93.4  82 | 140  112 | 3.35  2.78 | 3.56  2.88 | 2.0  1.8 |
| 5FA-noFA2 | 86  53 | -164  -176 | -128  -139 | 93.5  81.7 | 140  111 | 3.36  2.80 | 3.54  2.85 | 2.1  1.8 |
| 6FA | 89  51 | -164   -173 | -129   -139 | 93  80 | 140  112 | 3.32  2.82 | 3.57  2.89 | 2.1  1.7 |

**Table S3. Details on molecular composition and dimensions of model systems in the MD simulations.**

|  | **HSA residue range** | **palmitate number** | **Na^+^**  **number** | **Cl^-^** **number** | **water** **molecules** | **total**  **atoms** | **PBC box volume [nm^3^]** |
| --- | --- | --- | --- | --- | --- | --- | --- |
| noFA | 3-583 | 0 | 122 | 111 | 37093 | 37909 | 1195.5 |
| 5FA-noFA2 | 3-583 | 5 | 127 | 111 | 37027 | 37853 | 1195.2 |
| 6FA | 3-583 | 6 | 128 | 111 | 37012 | 37840 | 1195.9 |

**Table S4. Fitting approach used for ITC studies examining Zn^2+^ binding to rHA and H9A, H67A and H247 mutant albumins and resulting data.** Parameters that were fixed or varied (V) are indicated. The rHA fitted to a two-sets-of sites model where N1 and N2 were both fixed to 1. The data could also be fitted to a similar model where N2 was fixed to 2, but the fit was poorer. The H9A data fitted to a similar model where K_ITC2_ and ΔH2 were fixed based upon the values obtained for rHA. N2 was reduced (as expected) but N1 was also reduced to 0.51. Noteworthily, the difference in order of magnitude between the binding affinity of site A and B is less than 2 and this indicates overlapping equilibria which cannot be fully deconvoluted by data fitting, as noted previously (Sobczak et al., 2021). The H67A and H247A mutants were fitted to a two-sets-of-sites model where K_ITC1_ and ΔH1 were fixed based upon the values obtained for rHA. In each case (as expected) N1 was zero. However, there were observed increases in N2, possibly in part due to perturbation of site A such that Zn^2+^ may still bind to the remaining (non-mutated) ligands at site A but with reduced affinity. Data fitting from the three mutant proteins in the presence of 5 mol. eq. palmitate resulted in each case in N1 = 0. In the cases of H67A and H247A this would have anyhow been expected due to disruption of site A, but in the case of H9A, this indicates a FFA-mediated effect. Data were fitted using Origin v7.0 software.

| **Protein** | **Model** | **Fitting parameters** | | | | | | **χ^2^** |
| --- | --- | --- | --- | --- | --- | --- | --- | --- |
|  |  | N1 | K_ITC1_  (M^-1^) | ΔH1  (cal/mol) | N2 | K_ITC2_  (M^-1^) | ΔH2  (cal/mol) |  |
| rHA | Two-sets-of-sites | Fixed  1 | V  1000000 | V  -6032 | Fixed  1 | V  12000 | V  -12300 | 5863 |
| rHA | Two-sets-of-sites | Fixed  1 | V  1000000 | V  -6309 | Fixed  2 | V  13500 | V  -6500 | 44302 |
| H9A | Two-sets-of-sites | V  0.51 | Fixed  1000000 | Fixed  -6032 | V  0.34 | Fixed  12000 | Fixed  -12300 | 3650 |
| H67A | Two-sets-of-sites | V  0.00 | Fixed  1000000 | Fixed  -6032 | V  2.47 | V  59500 | V  -2103 | 5854 |
| H247A | Two-sets-of-sites | V  0.00 | Fixed  1000000 | Fixed  -6032 | V  1.66 | V  49700 | V  -2133 | 413 |
| H9A + 5 mol. eq. palmitate | Two-sets-of-sites | V  0.00 | Fixed  1000000 | Fixed  -6032 | V  2.53 | V  43000 | V  -1691 | 398 |
| H67A + 5 mol. eq. palmitate | Two-sets-of-sites | V  0.00 | Fixed  1000000 | Fixed  -6032 | V  1.96 | V  83800 | V  -2904 | 787 |
| H247A + 5 mol. eq. palmitate | Two-sets-of-sites | V  0.00 | Fixed  1000000 | Fixed  -6032 | V  0.86 | V  97600 | V  -1552 | 511 |

**Table S5.** **Fitting approach used for ITC studies examining myristate binding to HSA and Y150F/R257A/S287A albumin and resulting data.** Parameters that were fixed or varied (V) are indicated. Various 2 and 3 sets-of-sites models were attempted based upon the accepted view that HSA possesses 7 FFA binding sites (2-3 high affinity sites and several lower affinity sites). The HSA data could be fitted with two-sets-of-sites model with N1 fixed to 2 and N2 fixed to 5 (fit 1-1). The ^13^C-NMR data obtained as part of this study suggested that a high affinity site (the FA2 site) was absent in the Y150F/R257A/S287A mutant. Unfortunately, the Y150F/R257A/S287A data could not be fitted well (low GoF values) with a similar two-sets-of-sites model with N1 reduced to 1, whether K_ITC2_ and ΔH2 values for the second set of sites were fixed or not (fits 1-2 and 1-3). The HSA data could also not be fitted based upon a two-sets-of-sites models where N1 = 3 and N2 = 4 (fit-2-1). A reasonable fit was achieved for HSA using a three-sets-of-sites model, where N1 = 2, N2 = 1 or 2 and N3 = 4 or 3 (fits 3-1 and 3-2). The Y150F/R257A/S287A data could be fitted using a version of these model where N1 is reduced from 2 to 1, K_ITC1_ and ΔH1 values allowed to vary and all other parameters fixed to those obtained for HSA (fit 3-2). A better K_ITC1_ value was obtained using fit 3-3 when the N2 = 1 not 2 (fit 3-4). Data were fitted using AFFINIMETER software. GoF indicates goodness of fit, which is provided by AFFINIMETER to indicate reliability of the fit rather than a χ^2^ value.

| **Fit** | **Protein** | **Model** | **Fixed Parameters** | | | | | | | | | **GoF** |
| --- | --- | --- | --- | --- | --- | --- | --- | --- | --- | --- | --- | --- |
|  |  |  | N1 | K_ITC1_  (M^-1^) | ΔH1  (cal/mol) | N2 | K_ITC2_  (M^-1^) | ΔH2  (cal/mol) | N3 | K_ITC3_  (M^-1^) | ΔH3  (cal/mol) |  |
| 1-1 | HSA | Two-sets-of-sites | Fixed  2 | V  1070000 | V  -11611 | Fixed  5 | V  63700 | V  -2142 | - | - | - | 40.6% |
| 1-2 | Y150F/R257A/S287A | Two-sets-of-sites | Fixed  1 | V  2820000 | V  -22962 | Fixed  5 | Fixed  63700 | Fixed  -2142 | - | - | - | 18.8% |
| 1-3 | Y150F/R257A/S287A | Two-sets-of-  sites | Fixed  1 | V  80800 | V  -72531 | Fixed  5 | V  46000 | V  -8378 | - | - | - | 33.5% |
| 2-1 | HSA | Two-sets-of-sites | Fixed  3 | V  1740000 | V  -11611 | Fixed  4 | V  54200 | V  -100000 | - | - | - | 12.9% |
| 2-2 | Y150F/R257A/S287A | Two-sets-of-sites | Fixed  2 | V  4030000 | V  -8839 | Fixed  4 | V  9582 | V  -3365 |  |  |  | 57.0% |
| 3-1 | HSA | Three-sets-of-sites | Fixed  2 | V  1760000 | V  -10907 | Fixed  1 | V  391000 | V  -1928 | Fixed  4 | V  31100 | V  -3107 | 67.6% |
| 3-2 | HSA | Three-sets-of-sites | Fixed  2 | V  14900000 | V  -10926 | Fixed  2 | V  2190000 | V  -1245 | Fixed  3 | V  33200 | V  -3660 | 68.8% |
| 3-3 | Y150F/R257A/S287A | Three-sets-of-sites | Fixed  1 | V  16000000 | V  -8369 | Fixed  1 | V  1230000 | V  -10119 | Fixed  4 | V  81300 | V  -3329 | 63.5% |
| 3-4 | Y150F/R257A/S287A | Three-sets-of-sites | Fixed  1 | V  13200 | V  -7450 | Fixed  2 | V  6930000 | V  -8764 | Fixed  3 | V  199000 | V  -3221 | 67.2% |

**Table S6.** **Fitting approach used for ITC studies examining Zn^2+^ binding to rHA and Y150F/R257A/S287A albumin and resulting data.** Parameters that were fixed or varied (V) are indicated. The rHA data fitted better to a two-sets-of-sites model than a one-set-of-sites model, where the data suggested the involvement of a higher and lower affinity set of sites (incorporating sites A and B, respectively). The ΔH2 value was high, which may reflect that the stoichiometry of the lower-affinity set-of-sites may be larger than 1. Indeed, the existence of tertiary sites with very low affinity was previously suggested (Handing et al., 2016). Nonetheless, an attempt to fit the data with N2 fixed to 2 led to a fit (Entry 3; albeit with a large χ^2^). In the presence of 1 mol. eq. palmitate N1 reduced to 0.77 whereas N2 = 2.01 (Entry 4). Addition of further equivalents reduced N1 effectively to zero upon presence of 5 mol. eq. The Y150F/R257A/S287A mutant data also fitted to a two-sets-of-sites model with all parameters allowed to be varied, with a good fit achieved where N1 = 0.79. Unlike with the wildtype protein, the addition of palmitate did not affect N1. The N2 value was however seen to reduce at high FFA loading (4 and 5 mol. eq.). However, models with N2, K_ITC2_ and ΔH2 values fixed to those obtained in the absence of palmitate and the N1, K_ITC1_ and ΔH1 values allowed to vary produced reasonable fits. Data were fitted using Origin v7.0 software.

| **Protein** | **Model** | **Fitting parameters** | | | | | | **χ^2^** |
| --- | --- | --- | --- | --- | --- | --- | --- | --- |
|  |  | N1 | K_ITC1_  (M^-1^) | ΔH1  (cal/mol) | N2 | K_ITC2_  (M^-1^) | ΔH2  (cal/mol) |  |
| rHA | One-set-of-sites | V  1.85 | V  26500 | V  -9332 | - | - | - | 30739 |
| rHA | Two-sets-of-sites | V  1.15 | V  1000000 | V  -5978 | V  0.982 | V  10200 | V  -12110 | 4609 |
| rHA | Two-sets-of-sites | Fixed  1 | V  1000000 | V  -5978 | Fixed  2 | V  13400 | V  -6500 | 44326 |
| rHA + 1 mol. eq. palmitate | Two-sets-of-sites | V  0.77 | Fixed  1000000 | V  -5978 | V  2.01 | V  12200 | V  -6500 | 9315 |
| rHA + 2 mol. eq. palmitate | Two-sets-of-sites | V  0.57 | Fixed  1000000 | V  -5978 | V  2.02 | V  12600 | V  -6500 | 12697 |
| rHA + 3 mol. eq. palmitate | Two-sets-of-sites | V  0.17 | Fixed  1000000 | V  -5978 | V  1.73 | V  10700 | V  -8226 | 9879 |
| rHA + 4 mol. eq. palmitate | Two-sets-of-sites | V  0.10 | Fixed  1000000 | V  -5978 | V  1.81 | V  8830 | V  -7678 | 3399 |
| rHA + 5 mol. eq. palmitate | Two-sets-of-sites | V  0.01 | Fixed  1000000 | V  -5978 | V  2.35 | V  4820 | V  -6500 | 9753 |
| Y150F/R257A/S287A | Two-sets-of-sites | V  0.79 | V  1000000 | V  -6426 | V  1.72 | V  13000 | V  -6878 | 8250 |
| Y150F/R257A/S287A + 1 mol eq. palmitate | Two-sets-of-sites | V  0.78 | Fixed  1000000 | Fixed  -6426 | V  1.75 | V  16000 | V  -6500 | 10609 |
| Y150F/R257A/S287A + 2 mol eq. palmitate | Two-sets-of-sites | V  0.83 | Fixed  1000000 | Fixed  -6426 | V  1.69 | V  15300 | V  -6599 | 6242 |
| Y150F/R257A/S287A + 3 mol eq. palmitate | Two-sets-of-sites | V  0.78 | Fixed  1000000 | Fixed  -6426 | V  1.32 | V  11600 | V  -8325 | 5005 |
| Y150F/R257A/S287A + 4 mol eq. palmitate | Two-sets-of-sites | V  0.71 | Fixed  1000000 | Fixed  -6426 | V  0.72 | V  9510 | V  -17280 | 3486 |
| Y150F/R257A/S287A + 4 mol eq. palmitate | Two-sets-of-sites | V  0.79 | V  1140000 | V  -6187 | Fixed  1.72 | Fixed  13000 | Fixed  -6878 | 11880 |
| Y150F/R257A/S287A + 5 mol eq. palmitate | Two-sets-of-sites | V  0.75 | Fixed  1000000 | Fixed  -6426 | V  0.40 | V  8210 | V  -34060 | 3879 |
| Y150F/R257A/S287A + 5 mol eq. palmitate | Two-sets-of-sites | V  0.88 | V  1100000 | V  -6434 | Fixed  1.72 | Fixed  13000 | Fixed  -6878 | 3456 |

**Figure S2. Zn^2+^-binding to HSA and FA2-KO proteins loaded with palmitate. A**. ITC spectra showing Zn^2+^-binding to rHA loaded with 0-5 mol. eq. of palmitate (palm). **B**. ITC spectra showing zinc binding to the FA2-KO mutant albumin loaded with 0-5 mol. eq. of palmitate. Note that in contrast to the effects observed with the wildtype protein, Zn^2+^-binding at site 1 (site A) of FA2-KO appeared unaffected by binding of palmitate.

**Table S7.** Resulting measured parameters for the global fitting of Zn^2+^-binding to HSA and FA2-KO proteins loaded with palmitate (shown in Figure 4).

| **Sites per set** | **Degree of occupation** | **K**  **[M^-1^]** | **ΔH**  **(Kcal mol^-1^)** |
| --- | --- | --- | --- |
| 1 | Θ | 2.9 ± 0.1 × 10^5^ | -9.7 ± 0.1 |
| 2 | 1 | 0.25 ± 0.02 × 10^5^ | -3.74 ± 0.07 |

**Figure S3.** **Domain IA-IIA interface of unliganded HSA.** The positions of amino acid sidechains that provide ligands for Zn^2+^ at site A are stabilised by H-bonds involving Asn99, His247 and Asp249 (green dashed lines) and non-classical H-bonds involving Tyr30, His67, Asn99, His247, Gly248 and Asp249 (grey dashed lines). Diagram drawn using coordinates from PDB: 1BM0 (Sugio et al., 1999).

**Figure S4. Domain IA-IIA interface of HSA with palmitate bound.** The binding of palmitate at FA2 invokes a change in structure (relative to non-complexed albumin)**.** This arrangement is in part stabilised by an interdomain H-bond between Lys106 and Gly248 (green dashed line). Note that Asp249 and His67 could potentially co-ordinate Zn^2+^ with an additional ligand provided by either Asn99 or Glu252. Diagram drawn using coordinates from PDB: 1E7H (Bhattacharya et al., 2000).

**References**

Bhattacharya A.A., Grüne T., and Curry S. (2000) Crystallographic analysis reveals common modes of binding of medium and long-chain fatty acids to human serum albumin. *J. Mol. Biol.* 303, 721-732. <https://doi.org/10.1006/jmbi.2000.4158>.

Handing, K.B., Shabalin, I.G., Kassaar, O., Khazaipoul, S., Blindauer, C.A., Stewart, A.J., Chruszcz, M., and Minor, W. (2016) Circulatory zinc transport is controlled by distinct interdomain sites on mammalian albumins. *Chem. Sci.* 7, 6635-6648. <https://doi.org/10.1039/C6SC02267G>.

Sobczak, A.I.S., Katundu, K.G.H., Phoenix, F., Khazaipoul, S., Yu, R., Lampiao, F., Stefanowicz, F., Blindauer, C.A., Pitt, S.J., Smith, T.K., Ajjan, R.A., et al. (2021) Albumin-mediated alteration of plasma zinc speciation by fatty acids modulates blood clotting in type-2 diabetes. *Chem. Sci.* 12, 4079-4093. <https://doi.org/10.1039/D0SC06605B>.

Sugio, S., Kashima, A., Mochizuki, S., Noda, M., and Kobayashi, K. (1999) Crystal structure of human serum albumin at 2.5 Å resolution. *Protein Eng.* 12, 439-446.

<https://doi.org/10.1093/protein/12.6.439>.
